# Recent Trends in Adipose Tissue-Derived Injectable Therapies for Osteoarthritis: A Scoping Review of Animal Models

**DOI:** 10.3390/medicina60050707

**Published:** 2024-04-25

**Authors:** Alina Otilia Adam, Horea Rares Ciprian Benea, Horia Mihnea Fotescu, Miriam Alcalá Ruiz, George Claudiu Cimpean, Vladimir Ciornei, Arsenii Cernacovschi, Andrei Rares Edves, Maria Crisan

**Affiliations:** 1 Department of Orthopedics and Traumatology, “Iuliu Hatieganu” University of Medicine and Pharmacy, 400132 Cluj-Napoca, Romania; adam.alina.otilia@elearn.umfcluj.ro (A.O.A.);; 2 Faculty of Medicine, “Iuliu Hatieganu” University of Medicine and Pharmacy, 400347 Cluj-Napoca, Romania; 3 Department of Histology, “Iuliu Hatieganu” University of Medicine and Pharmacy, 400349 Cluj-Napoca, Romania; maria.crisan@umfcluj.ro

**Keywords:** osteoarthritis, animal model, adipose tissue-derived injectable therapies, stromal vascular fraction, fragmented adipose tissue

## Abstract

*Background and Objectives*: This scoping review investigates recent trends in adipose tissue-derived injectable therapies for osteoarthritis (OA) in animal models, focusing on minimally manipulated or lightly processed adipose tissue. By evaluating and examining the specific context in which these therapies were investigated across diverse animal OA models, this review aims to provide valuable insights that will inform and guide future research and clinical applications in the ongoing pursuit of effective treatments for osteoarthritis. *Materials and Methods*: This research conducted a comprehensive literature review of PubMed and Embase to determine studies about minimally manipulated adipose tissue-derived injectable therapies for osteoarthritis investigated using animal models. The primary search found 530 results. After excluding articles that focused on spontaneous osteoarthritis; on transfected, preconditioned, cultured, or co-cultured adipose-derived stem cells; and articles with unavailable full text, we included 11 articles in our review. *Results*: The examined therapies encompassed mechanical micro-fragmented adipose tissue (MFAT) and stromal vascular fraction (SVF) obtained via collagenase digestion and centrifugation. These interventions were evaluated across various animal models, including mice, rats, rabbits, and sheep with induced OA. Notably, more studies concentrated on surgically induced OA rather than chemically induced OA. The assessment of these therapies focused on elucidating their protective immunomodulatory, anti-inflammatory, and chondroregenerative potential through comprehensive evaluations, including macroscopic assessments, histological analyses, immunohistochemical examinations, and biochemical assays. *Conclusions*: This review provides a comprehensive analysis of adipose tissue-derived injectable therapies for osteoarthritis across diverse animal models. While revealing potential benefits and insights, the heterogeneity of data and the limited number of studies highlight the need for further research to formulate conclusive recommendations for clinical applications.

## 1. Introduction

Osteoarthritis (OA) is a complex disease characterized by degenerative changes that involve all tissues within the joint structure [1]. The prevalence of OA, though it varies based on geographic location, race, age, gender, obesity, or altered joint mechanics, is a significant public health concern, particularly among middle-aged and elderly individuals. This condition leads to substantial pain, disability, and reduced quality of life and therefore impacts disability and healthcare costs globally [2]. Recent studies have underlined the involvement of immune cells in both the development and progression of osteoarthritis [3]. Inflammatory components, such as cytokines and chemokines, are synthesized by chondrocytes and synoviocytes within the affected joints of osteoarthritis patients. Additionally, synovial fibroblasts contribute to this inflammatory milieu by secreting proinflammatory cytokines and matrix-degrading enzymes under osteoarthritic conditions. These inflammatory mediators alter cell signaling pathways, gene expression, and the behavior of joint tissue, disrupting the homeostatic balance between degradation and repair mechanisms in the articular cartilage [4]. Ultimately, these cascading events disrupt the anatomical and physiological functions of the joint [5]. The cellular and molecular mechanisms of OA initiation and progression are incompletely understood, necessitating the development of novel targeted and effective disease-modifying treatments. The development of OA is influenced by various risk factors that impact the articular cartilage and subchondral bone, which makes the accurate study of this disease through in vitro models alone challenging [6,7]. Therefore, it is necessary to establish validated in vivo models to investigate OA pathology effectively and assess potential treatments. Various animal models have been created to investigate the development of OA and evaluate the effectiveness of new diagnostic and therapeutic approaches. However, due to the heterogeneity of this disease, there is not a single animal model that can fully reflect the onset and progression of OA in humans. Therefore, model selection is based on the specific etiology under investigation and the intended purpose, highlighting the need for a systematic approach to evaluate and compare the efficacy of different animal models for studying OA.

In recent years, mesenchymal stem cell (MSC)-based therapies for cartilage lesions and OA have shown promise [8]. MSCs are versatile progenitor cells capable of self-renewal and differentiation into various cell types, including adipocytes, osteoblasts, and chondrocytes. Bone marrow-derived mesenchymal stem cells (BMSCs) have been the subject of extensive research in animal models and some clinical cases for their potential in OA treatment [9]. However, adipose-derived stem cells (ADSCs) have gained attention due to their abundance and ease of isolation compared to BMSCs. ADSCs are readily cultured and proliferate more rapidly than BMSCs, making them a more accessible source of stem cells [10]. Additionally, ADSCs exhibit less age-related telomerase decline than BMSCs [11]. Moreover, the immunoregulatory and anti-inflammatory properties of these cells, mediated by paracrine factors, position MSC-based therapies as promising candidates for OA treatment [12].

This scoping review aims to thoroughly evaluate the recent research trends in the treatment of OA in animal models using adipose tissue-derived injectable therapies, with a focus on two distinct preparation methods: the utilization of mechanically minimally manipulated adipose tissue and the employment of adipose-derived tissue that has undergone light processing after harvest thereby preserving its intrinsic, unaltered characteristics [13,14]. This assessment aims to synthesize the current body of evidence to assess the effectiveness of these therapies in the context of different animal osteoarthritis models, providing insights that can guide future research and clinical applications in this area. Furthermore, this scoping review aims to serve as a reference for further studies to develop improved therapies for osteoarthritis.

## 2. Materials and Methods

### 2.1. Study Selection

To fulfill the objectives of this investigation, the research question was formulated using the PICO framework (Population, Intervention, Comparison, Outcome) [15]. This study aimed to investigate the use of minimally manipulated adipose tissue-derived injectable therapies (I) in animal models with induced knee or hip osteoarthritis (P), comparing them with untreated joints or other treatment options (C) to better understand osteoarthritis progression in different contexts (O).

### 2.2. Inclusion Criteria

The inclusion criteria encompassed studies that reported cases of osteoarthritis in animal models induced through mechanical, surgical, and chemical methods. Specifically, only studies on knee and hip osteoarthritis that focused on minimally manipulated adipose tissue-derived injectable therapies were considered. Additionally, only English-language articles were included in the screening process.

### 2.3. Exclusion Criteria

The exclusion criteria included studies focusing on spontaneous osteoarthritis animal models, human models of osteoarthritis, and other forms of arthritis. Studies that utilized genetic manipulations to induce osteoarthritis in animal models were also excluded. Moreover, a timeframe spanning the past decade was adopted as the framework for consideration. Consequently, studies conducted before 2013 were excluded from evaluation, ensuring the focus remained on the most recent literature. Additionally, technical notes, letters to editors, instructional courses, studies with specific therapeutic purposes, drug tests, pilot studies, and studies with a sample size of fewer than five animals per group were deemed ineligible for the current research in order to provide more reliable evidence for the evaluation of therapeutic interventions. Moreover, studies involving cultured or co-cultured, transfected, preconditioned ADSCs were not selected, as this review aimed to highlight treatment approaches that preserve the intrinsic characteristics of the adipose tissue. This focus aligns with the objective of evaluating therapies that are minimally manipulated.

### 2.4. Search

A scoping literature review following the Preferred Reporting Items for Systematic Reviews and Meta-Analyses (PRISMA) extension for Scoping Reviews (PRISMA-ScR) [16] guidelines was conducted on PubMed and Embase to investigate the intra-articular use of adipose tissue-derived therapies for osteoarthritis. The search was limited to English publications, and two authors performed the searches.

The search spanned from January 2013 to December 2023. It utilized the following search string: (adipose-derived OR micro-fra* adipose tissue OR microfra* adipose tissue OR fra* adipose tissue OR stromal vascular fraction OR SVF) AND (osteoarthritis). An independent manual search was also carried out with adapted terms on the database and on the reference lists of relevant review studies.

### 2.5. Methodology for Data Collection

Two researchers independently executed the data collection procedure, with a third reviewer (H.R.C.B.) enlisted to arbitrate in the event of any discrepancies. The screening protocol involved a sequential process initiated by A.O.A. and M.A.R., beginning with evaluating titles and abstracts, followed by a comprehensive examination of the full-text versions. Articles that withstood the initial rejection based on titles and abstracts underwent thorough reading in their entirety. In instances of discord, H.R.C.B. facilitated resolution. The enumeration of included and excluded publications was documented through adherence to the PRISMA flowchart [17].

### 2.6. Data Elements

Various data points encompassing primary author details, publication year, animal species, sample size, type of induction and classification (mechanical, surgical, and chemical), investigated joint, treatment modalities, follow-up duration, results, and characteristics of adipose-based therapies (including source, dose, additional procedures, injective protocol, and processing modality) were systematically extracted. A comprehensive synthesis was conducted to analyze the disease-modifying impacts resulting from the intra-articular application of various preparations in different OA contexts. This analysis was based on evaluating objective evidence measures of effect, including imaging, macroscopic assessment, histological examination, immunohistochemical analysis, and biochemical and molecular biology measurements with a focus on osteoarthritis processes.

### 2.7. Evaluation of Biases

**Figure 1:**
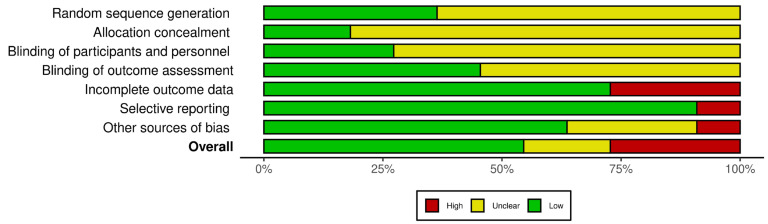
SYRCLE’s risk of bias tool assessment of the included studies. The bar chart illustrates the proportion of studies meeting each quality criteria.

The potential biases within the included articles were appraised using the Systematic Review Centre for Laboratory Animal Experimentation (SYRCLE) tool [18]. This tool, an adapted version of the Cochrane Collaboration RoB Tool, encompasses ten items addressing various biases such as selection, performance, detection, attrition, reporting, and “other” biases. The assessment of each item involved is illustrated in Figure 1. The main sources of bias that were identified include the lack of explicit documentation related to the random allocation of animals into experimental groups. There was insufficient clarity regarding the blinding procedures during outcome assessments despite the involvement of multiple researchers. Additionally, some studies exhibited high bias in outcome data reporting due to the partly descriptive nature of the results without adhering to standardized scoring methodologies.

## 3. Results

### 3.1. Selection of Studies

**Figure 2:**
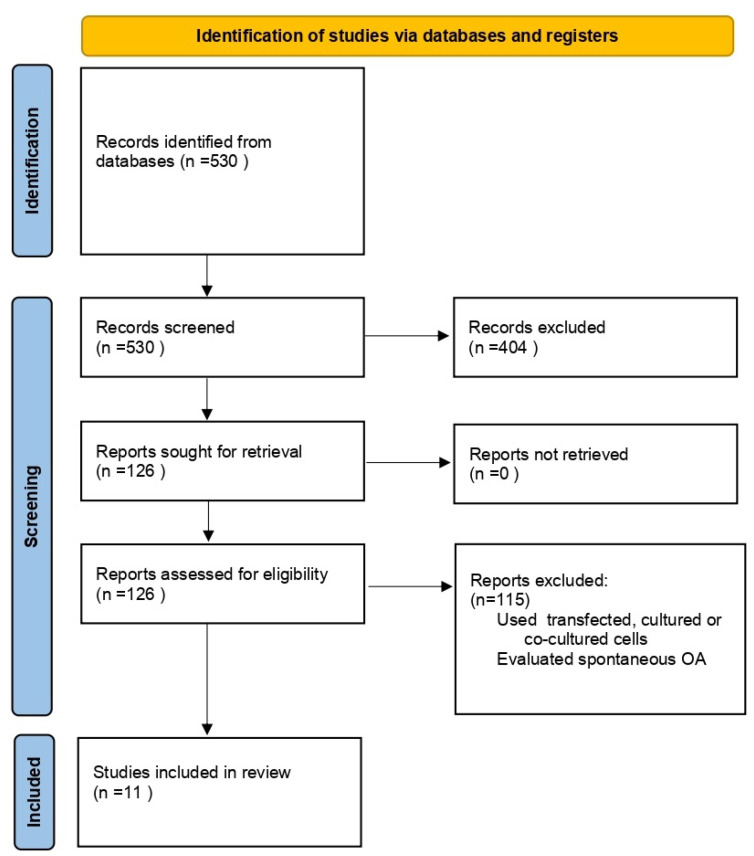
Prisma flow diagram.

In total, 530 articles were initially identified in the search of pertinent literature. Upon screening titles and abstracts, 404 out of the 530 papers were excluded (used different types of transport media to enhance adipose tissue-derived therapies, animal clinical trials, pilot studies, and review articles). Subsequently, 126 articles underwent a full-text review, with 115 excluded due to differing cell sources; use of transfected, cultured, or co-cultured cells; or the analysis of spontaneous osteoarthritis. Consequently, 11 articles successfully passed the final screening and were included in the analysis. Figure 2 illustrates the screening process.

### 3.2. Characteristics of Selected Studies

The 11 selected articles involved a total of 336 animals recruited for the investigation. Within this cohort, two studies reported details on chemically induced osteoarthritis [19,20], while nine focused on osteoarthritis induction through surgical interventions [21,22,23,24,25,26,27,28,29]. Among these studies, four documented treatments as exclusively autologous [20,23,26,28], and two as purely xenogeneic [19,21], utilizing ADSCs from human origins (hADSCs). Additionally, in five studies, treatments involved a combination of autologous and allogeneic approaches [22,24,25,27,29]. Notably, there were no instances where the treatment regimen was exclusively allogeneic. The studies are summarized in chronological and animal size order in Table 1 and Table 2.

### 3.3. Animal Models

Among the 11 chosen studies, various animal species were employed in the evaluation of osteoarthritis, with sheep emerging as the predominant model. Four authors specifically opted for male and female sheep [22,23,24,25]. However, seven authors chose to investigate and induce osteoarthritis in alternative animal species: mice, rats, and rabbits [19,20,21,26,27,28,29]. 

### 3.4. Type of Joint

During their study, the researchers targeted a specific joint in different animal models, which resulted in the knee joint being the most commonly selected joint for inducing OA. Specifically, among the selected studies, two opted for knee OA induction in mice [19,21], two in rats [20,29], three in rabbits [26,27,28], and four in sheep [22,23,24,25].

### 3.5. Induction Methods

Among the two studies outlining OA induction in animals through a chemical approach, one employed intra-articular injection of collagenase II with CaCl_2_ in mice [19], while the other utilized intra-articular injection of sodium iodoacetate in rats [20]. 

In contrast, among the nine studies concentrating on the surgical approach for inducing OA in animals, one adopted surgical destabilization through anterior cruciate ligament transection (ACLT) in addition to medial meniscectomy (MMx) [23], while another concentrated on anterior cruciate ligament, medial meniscus, and medial collateral ligaments transection [29]. Six studies exclusively conducted either ACLT [26,27,28] or MMx [22,24,25]. One study explored a model of osteoarthritis induced by resecting the medial meniscotibial ligament to destabilize the medial meniscus [21]. Six studies induced OA in bilateral knees [19,22,24,25,26,27], one study in the left knee [20], three studies in the right knee [21,23,29], and one study did not clearly specify the investigated knee for inducing OA [28].

### 3.6. Analysis of Adipose Tissue-Derived Injectable Therapies

#### 3.6.1. Adipose Tissue-Derived Injectable Therapy Type

Three studies directed their attention to the utilization of minimally manipulated micro-fragmented adipose tissue (MFAT) as follows: two studies employed exclusively mechanical and filtering devices [26,27], while one chose to enhance the process with centrifugation [29]. Additionally, eight studies scrutinized the application of adipose tissue-based therapies that underwent light processing of the tissue after harvest with collagenase digestion and centrifugation to obtain stromal vascular fraction (SVF) [19,20,21,22,23,24,25,28].

#### 3.6.2. Therapy Administration Timing and Frequency Following OA Induction

The timing of therapy administration after OA induction varied depending on the OA model and the aspect of the disease intended to be analyzed, early OA or progressive OA. In the two studies of the mice OA model, the chosen therapy was injected immediately after surgical intervention inducing OA [21] or 5 days after induction [19], respectively. In the sheep model, three studies injected the therapy at 6 weeks [22,24,25], and one administered it at 12 and 15 weeks [23] after OA induction. All three studies on the rabbit model injected the therapy at 8 weeks [26,27,28] after OA-inducing surgery. Regarding the rat model OA, one study injected the therapy two weeks after OA induction [20] and one study administered it weekly for six weeks [29].

### 3.7. Evaluation of Adipose Tissue-Derived Injectable Therapies

#### 3.7.1. Time of Evaluation after Administration of Injectable Therapy

In the murine OA model, the evaluation of therapeutic outcomes after adipose tissue-derived injectable therapies took place four weeks post-treatment [19]. Another study within the murine OA model assessed half of the population included in the study at four weeks and the remaining half at eight weeks after treatment [21]. Within the investigations focusing on the sheep OA model, two studies examined therapeutic outcomes at the three-month mark post-injection [22,23], while two additional studies assessed outcomes at both the three-month and six-month intervals post-treatment [24,25]. Each study involving the rabbit OA model conducted a dual analysis of therapeutic outcomes at two and four months [26], at two and three months [27], or one week and one month post-treatment [28]. 

#### 3.7.2. Macroscopic Evaluation

In three distinct animal models of OA, the assessment of treatment efficacy involved macroscopic evaluations. The evaluation criteria varied across species: the sheep model studies employed a gross articular damage score and International Cartilage Research society score (ICRS) [30] to characterize articular appearance [22,23,24,25], rabbit model studies utilized either the Osteoarthritis Research Society International’s (OARSI) cartilage histopathology assessment system [28,31] or the macroscopic Hanashi score [31] for quantification [26], and rat studies employed a descriptive evaluation approach [20].

#### 3.7.3. Histologic Evaluation

The histological assessment of treatment efficacy was quantified by employing either the OARSI [32] or Laverty score [31] for cartilage evaluation, the Krenn [33] or Laverty score for synovium [31] characterization, and, in a singular study, the modified Pauli’s score [34] for meniscus analysis [27].

#### 3.7.4. Immunohistochemical Evaluation

Immunohistochemistry was employed to investigate the expression of OA-related proteins in the cartilage and macrophage-associated markers in the synovium. Specifically, eight studies assessed the expression of collagen type II [20,21,24,25,26,28], collagen type I [25,35], or collagen type X [23]. Additionally, five studies scrutinized the expression of matrix metalloproteinase-13 (MMP-13) [21,25,28], matrix metalloproteinase-1 (MMP-1) [26], disintegrin and metalloproteinase with thrombospondin motifs 5 (ADAMTS-5) [21], interleukin-6 (IL-6) [21], interleukin-1 beta (IL-1β) [21,25], SRY-box transcription factor 9 (SOX-9) [19,28], and tumor necrosis factor alpha (TNF-α) [26].

#### 3.7.5. Biochemical Evaluation

In the 11 studies selected, four [22,23,24,25] specifically delved into the outcomes of adipose tissue-derived therapy employing enzyme-linked immunosorbent assay (ELISA) analysis to assess various parameters, including IL-1β, cross-linked C telopeptide of type II collagen (CTX2), IL-6, TNF-α, and prostaglandin E2 (PGE2). The ELISA assessment enabled a precise and quantitative analysis of the levels of these crucial markers in both cartilage and synovial tissue. 

### 3.8. Articular Effects of Adipose Tissue-Derived Injectable Therapies

Administration of adipose tissue-derived therapy utilizing SVF in mice subjected to surgically induced OA-attenuated disease progression. A notably lower histological OARSI score was evident in the treated group compared to the control group that received phosphate-buffered saline injections [21]. Immunohistochemical analysis revealed a noteworthy increase in the number of type II collagen-positive cells in the treated group, while the number of chondrocytes expressing MMP-13, ADAMTS-5, IL-6, and IL-1β was significantly lower in comparison to the control group at both 4- and 8-weeks post-treatment. In mice with chemically induced OA [19], adipose tissue-derived therapy utilizing cultured suprapatellar fat pad hADSCs demonstrated a notable regenerative effect on joint structures, as indicated by the quantification of the histological OA damage score. The administration of hADSCs consistently led to structural regeneration, mitigating the loss of cartilage compared to the SVF and control groups. Immunohistochemical assessment of SOX-9, a chondrocyte progenitor marker, revealed that only the expanded hADSCs were capable of inducing efficient regeneration one month after treatment, displaying a significant increase in SOX-9 expression within the developing cartilage. A small proportion of SOX-9-positive cells exhibited co-staining with specific immunodetection for human cells, suggesting an endogenous induction of cartilage repair through hASC injection. In mice with chemically induced OA, characterized by aggressive joint degeneration, the injection of hADSCs proved more effective in reducing and restoring the functional anatomy of the intraarticular space in comparison to SVF.

In the surgically induced OA sheep model, the therapeutic efficacy of SVF was evaluated in comparison to culture-expanded hADSCs, saline (control), allogenic culture-expanded amniotic endothelial cells (AECs) or allogenic culture-expanded amniotic epithelial stem cells (AESCs). One study demonstrated the superiority of SVF-based therapy over the other interventions at the 6-month mark, promoting cartilage regeneration and mitigating the inflammatory microenvironment. SVF treatment showed superior effectiveness compared to AESC treatment, which, in turn, exhibited superiority over ADSC treatment. SVF outperformed AESCs concerning macroscopic scores at three months, Krenn scores at six months, and histologic OARSI scores at three and six months. At both the 3-week and 6-week evaluation stages, the percentage of cells expressing COL1A1 (Collagen Type I Alpha 1 Chain gene) and MMP-13 was significantly lower, while that of cells expressing COL2A1 (Collagen Type II Alpha 1 Chain gene) was significantly higher in the SVF-treatment group compared to AESC, ADSC, and control groups [25]. The levels of pro-inflammatory cytokines (CTX2, PGE2, IL-1β) in synovial fluid, as assessed by ELISA, exhibited decreased values in the SVF-treated group. Significantly lower levels of TNF-α and IL-6 were observed in both SVF- and AESC-treatment groups compared to the control and ADSC groups [22]. In the investigation evaluating the therapeutic efficacy of SVF, AECs, and ADSCs concerning articular cartilage viscoelastic parameters, notably the elastic modulus and cartilage relaxation time, SVF exhibited pronounced effectiveness. Comparative analysis with the other treatments revealed SVF’ superior success in attenuating the deterioration of articular cartilage viscoelastic parameters at 3 and 6 months post-treatment [24]. In comparing the impact of hyaluronic acid (HA) intra-articular injection in conjunction with varied doses of ADSCs or SVF, it was noted that high-dose ADSCs combined with HA significantly suppressed the expression of IL-1β and IL-6 in synovial fluid. In contrast, there were no statistically significant differences observed in the treatment combinations of low-dose ADSCs with HA and SVF with HA [23].

In the surgically induced OA lapine model, the therapeutic efficacy of SVF was investigated, comparing it with a control group receiving an intra-articular injection of serum albumin. At 8 and 12 weeks after OA onset, the treated group showed significantly lower OARSI scores and less cartilage damage than the control group, according to both macroscopic and histological assessments. Immunohistochemically, the SVF group showed a significantly lower proportion of MMP-13-positive cells and a significantly higher percentage of type II collagen-positive areas than the control group. Additionally, the proportion of SOX-9-positive cells was significantly higher in the SVF group than in the control group [28]. Comparing expanded ADSCs, SVF, and MFATs as intra-articular injectable therapies, notable differences in cartilage scores were observed at day 7 and day 30 of the assessment. Specifically, on day 7, SVF treatments exhibited a significantly lower score compared to MFAT and ASC treatments. By day 30, MFAT treatments showed a significantly higher score than SVF and ASC treatments. Notably, both SVF and MFAT groups displayed time-dependent effects on cartilage [27]. In another study that evaluated the same treatment options at 2- and 4-months follow-up revealed that MFAT showed the best results both in terms of qualitative and semi-quantitative evaluations of articular cartilage, with a more uniform staining, a smoother surface, and a significantly better Laverty score [26]. A lipophilic fluorescent membrane dye was employed for labeling to assess the spatial distribution of treatment cells following intra-articular injection. Notably, diverse migration patterns were discerned at 7- and 30-days post-injection for MFAT, SVF, and expanded ADSCs. Specifically, the MFAT group displayed lower cell migration in the synovial membrane than SVF and ASC groups on day 7 but exhibited higher migration to the synovial membrane than SVF and ASCs on day 30 [27]. 

In the rat model of chemically induced OA, both intra-articular SVF and expanded ADSC therapies exhibited, upon histological evaluation, a discernible thick layer of cartilage with a regular surface resembling that of normal cartilage. Both therapeutic interventions also significantly reduced plasma IL-1β levels on days 7 and 14 after treatment compared to the control group [20]. In the surgically induced OA rat model, the treated group received weekly injections of micro-fragmented and centrifuged adipose tissue for six weeks. This group exhibited significantly lower modified Mankin histologic scores compared to the control group at 2 and 6 weeks following OA induction [29].

## 4. Discussion

The particularity of this scoping literature review lies in its comprehensive analysis of trends related to adipose tissue-derived injectable therapies for osteoarthritis, with a specific focus on two distinct preparation methods: the use of minimally manipulated adipose tissue and the application of adipose-derived tissue subjected to light processing post-harvest. However, what sets this review apart is its unique approach to analyzing these therapeutic modalities in the context of various types of animal osteoarthritis models. Rather than exclusively focusing on clinical studies or human trials, this review systematically considers evidence from different animal models, encompassing diverse species. By examining the therapeutic outcomes and trends in these varied animal models, the review aims to provide a nuanced understanding of the potential effectiveness of adipose tissue-derived injectable therapies in addressing the pathogenesis and progression of osteoarthritis. This distinctive approach enhances the generalization of findings, offering valuable insights that can guide future research and clinical applications in OA.

OA is a complex joint disorder affecting all tissues within and surrounding the joint. Fundamental manifestations include articular cartilage degradation, subchondral bone thickening, osteophyte formation, and variable degrees of synovial inflammation. Highly regulated anabolic and catabolic mechanisms maintain and adapt cartilage behavior to disruptive factors [36]. OA pathogenesis involves cartilage breakdown and joint systemic structural changes, with low-grade chronic joint inflammation emerging as a central role in determining OA pathophysiology. Innate and adaptive immunological mechanisms drive the progression of OA. Dysregulation by various biofactors disrupts cartilage homeostasis. Chondrocytes, synoviocytes, and osteoblasts generate pro-inflammatory mediators such as cytokines and reactive oxygen species [37]. These mediators promote the degradation of the extracellular matrix, loss of cartilage, fibrillation, erosion, cell death, matrix calcification, and vascular invasion. Chondrocyte activities, including proliferation, matrix deposition, inflammatory cytokine production, and response to signaling molecules are altered during OA progression, contributing to phenotypic changes [36]. 

Age-associated chondrocyte senescence exacerbates cartilage degeneration, driven by elevated expression of detrimental factors, including IL-1β, IL-7 and MMP-13. Trauma to articular cartilage results in cell loss, reactive oxygen species production, and proteolytic enzyme release, further exacerbating cartilage degradation. Obesity contributes to OA through mechanical loading and metabolic and inflammatory mediator release, inducing cartilage matrix damage and subchondral bone remodeling. Physiological mechanical loading maintains cartilage homeostasis by suppressing pro-inflammatory cytokines, enhancing anti-inflammatory signaling, and reducing matrix-degrading enzyme activity. However, supraphysiological loading shifts the balance toward catabolic processes, promoting cartilage defects, bone marrow lesions, subchondral sclerosis, and OA onset. Abnormal mechanical stress increases pro-inflammatory mediator production, further disrupting cartilage metabolism [36]. The multifactorial nature of osteoarthritis highlights the importance of exploring various therapeutic avenues targeting inflammatory, metabolic, and mechanical pathways to effectively mitigate disease progression.

The choice of animal models for OA research depends on various factors, including the specific research question, the desired outcome measures, and the availability of resources. For example, mouse and rat models are commonly used due to their relatively low cost, ease of handling, and the availability of genetically modified strains that mimic specific aspects of human OA. On the other hand, large animal models such as dogs or horses are preferred when studying the efficacy and safety of potential therapies, as their joint size and biomechanics more closely resemble those of humans [38]. 

It is vital to consider the differences between species, as they can significantly impact the phenotype of osteoarthritis (OA) and how research findings are translated. For example, certain species, such as adult mice and rats, do not express key human OA factors such as MMP-1 and have variations in the structure of cartilage proteoglycans. This could affect enzyme activity and kinetics. Furthermore, cartilage thickness decreases as the size of the animal decreases. Differences in cartilage thickness related to animal size may impact the diffusion and penetration of compounds used in the induction or treatment of OA. When studying joint biomechanics, it is essential to consider the anatomical and structural differences. For instance, quadrupeds have knee joints with a greater tibial slope than humans, which can direct affected joint regions more posteriorly, especially in ACLT models. The extent of meniscal coverage and composition also varies across different species, which may impact the progression and size of osteochondral lesions. Additionally, the primary compartment or region of loading differs between humans and animals, such as the medial tibiofemoral in human knees and lateral tibiofemoral in rabbits, which can directly influence the most affected joint regions [39]. 

Studies have shown that isolated loss of ACL function in sheep and goats induces only mild osteoarthritis even with a prolonged time course. However, in same conditions, dogs exhibit progressive OA, characterized by cartilage erosion, subchondral bone resorption, sclerosis, and marginal osteophyte development. In mice, rapid and marked posterior tibial erosive disease has been observed in knee joints with dysfunction of the ACL [39]. 

When interpreting animal study results, it is essential to consider the differences between humans and experimental species. However, by studying and understanding these inherent differences, we can identify new pathways that may be exploited for treatment.

The choice of induction method can significantly impact the relevance and translatability of research findings to human clinical outcomes. Surgical induction is one of the most commonly used methods for inducing osteoarthritis in animal models. This method involves creating controlled joint instability or trauma, most frequently through ACLT or meniscectomy, to mimic the initiation and progression of osteoarthritis. This method enables the study of osteoarthritis development from its early stages to advanced pathology, allowing for comprehensive assessment of interventions at various disease stages. As a disadvantage, surgical induction involves invasive procedures that may introduce confounding variables and increase the risk of complications [40]. Another approach to inducing osteoarthritis in animal models is using chemical inducers, such as monosodium iodoacetate or papain. These substances are injected into the joint to disrupt cartilage integrity and induce degenerative changes characteristic of osteoarthritis. Chemical induction allows for precise control over the timing and extent of disease initiation, enhancing experimental reproducibility [1]. Unlike surgical induction, chemical induction can be performed without creating structural damage to the joint, reducing the risk of procedural complications [7]. While effective in inducing cartilage degeneration, chemical inducers may not fully replicate the etiological factors contributing to human osteoarthritis. Chemical inducers, by altering the joint environment, may influence researched therapies or have systemic effects beyond the targeted joint, introducing confounding variables unrelated to osteoarthritis pathology [41]. Mechanical induction involves applying mechanical stress or loading to the joint non-invasively to initiate joint degradation in a more controlled and accurate manner, replicating human damage [42]. Mechanical force is applied externally to the joint to cause harm to the bones, cartilage or soft tissues. This method includes techniques such as tibial compression and joint immobilization, which induce progressive cartilage degeneration and osteoarthritis-related joint changes [42]. Mechanical induction replicates the physical forces and stresses that contribute to joint degeneration in human osteoarthritis, enhancing the translational relevance of research findings. Researchers can adjust the magnitude and frequency of mechanical loading to study the impact of different loading patterns on osteoarthritis progression [7]. As drawbacks, the utilization of mechanical induction demands specialized equipment and expertise, thereby introducing technical intricacies into experimental procedures. Moreover, variations in the application of mechanical forces and disparities in animal joint responses can introduce variability in the study outcomes [1]. The reviewed articles demonstrate a preference for the surgical induction method in osteoarthritis research. This tendency highlights the importance of evaluating the pros and cons of these methods in relation to research objectives.

Currently, the management of OA focuses only on improving its symptoms and does not have any interventions to prevent or slow down the progression of OA. These therapies do not help regenerate damaged articular cartilage but aim to reduce pain and improve joint function [43]. Initially, OA is managed conservatively with exercises, weight loss, and physiotherapy, followed by paracetamol or non-steroidal anti-inflammatory drug administration for symptom control [44]. Intra-articular corticosteroid injections or hyaluronic acid viscosupplementation are utilized if conservative measures fail, with joint replacement surgery being the gold-standard option for end-stage OA [45].

In recent years, there has been a significant evolution in the field of regenerative medicine and tissue-based therapies for osteoarthritis [46]. Initially, considering cartilage regeneration, there was a shift from BMSCs therapy to utilizing ADSCs, followed by a subsequent progression towards therapies involving minimal processing of adipose tissue [47]. Several factors have driven the transition towards adipose tissue-derived therapies. Adipose tissue, readily accessible through minimally invasive procedures such as liposuction, has gained prominence due to its abundance and ease of extraction. Moreover, ADSCs exhibit properties comparable to those derived from other tissues, making them a versatile and promising source for regenerative therapies [9,48]. ADSCs have been observed to possess enhanced anti-inflammatory characteristics when contrasted with BMSCs, generating notably higher quantities of IL-1β receptor antagonist and the tissue-protective protein Tumor Necrosis Factor Stimulated Gene-6 (TSG-6). When evaluated for their role in OA, ADSCs displayed adaptability within the environment and demonstrated anti-inflammatory effects on chondrocytes and synoviocytes via the secretion of PGE2 [44]. The appeal extends to the cost-effectiveness of minimally manipulated adipose tissue-derived therapies, which do not require cell separation or culturing [49]. This economic advantage, coupled with the similarity in properties to other MSC sources, underscores the growing interest in adipose-derived therapies in regenerative medicine [13]. SVF and MFAT stand out as commonly cited therapies derived from minimally manipulated adipose tissue [13,14]. The initial procedure for isolating SVF from adipose tissue involved isolating adipocytes and stromal cells using collagenase [50]. Enzymatic digestion methods, commonly used to break down adipose tissue, are often employed when the aim is mesenchymal cell culture and expansion. Collagenase efficiently separates fat into two distinct layers: a floating fraction of mature adipocytes and a lower aqueous portion of cellular components, which can then be further separated through centrifugation [50]. Although an effective tool for SVF extraction, the potential trace amounts of residual collagenase in injectable products are highly detrimental to patient safety and can lead to adverse reactions [50]. In order to reduce the risk of residual enzyme contamination, one approach that has been proposed is mechanical disruption and filtration of adipose tissue to obtain a cell-rich fraction of MFAT [51]. This method has shown promising results in maintaining the inherent regenerative properties of adipose tissue while minimizing the risk of enzyme contamination [51]. One of the considered limitations of utilizing mechanical means to extract cells from adipose tissue was the relatively low cell yield when compared to enzymatic digestion protocols. This limitation arises from the strong bonds established between adipocytes and collagen, which resist disruption via mechanical means. However, adipose tissue can undergo filtration and emulsification facilitated by “nano-filters”, thereby providing a route for overcoming the limitation associated with mechanical means of extraction [50]. Moreover, MFAT is different from SVF in that it retains the intact microvascular structure of the extracellular matrix and provides a natural niche for various bioactive cellular subsets [52]. This enables a broader range of biological functions, including cell migration and modulation, as well as cell signaling, interaction, and differentiation [52]. Understanding the role of minimally processed adipose tissue therapies in facilitating regenerative processes is crucial for maximizing its therapeutic potential in treating osteoarthritis. Research efforts have increasingly focused on understanding the paracrine and immunomodulatory functions of the cellular components in the SVF and MFAT [46]. For instance, ADSC and endothelial progenitor cell components of minimally manipulated adipose tissue have been found to secrete an array of bioactive molecules, including growth factors and cytokines, which play a pivotal role in modulating the local tissue environment, encouraging angiogenesis, and regulating immune responses, thus activating an anti-inflammatory and pro-regenerative processes [53]. Furthermore, these cells can interact with resident cells in the joint, contributing to tissue repair and regeneration [53].

According to the reviewed studies, minimally manipulated adipose-derived therapies have consistently shown positive therapeutic outcomes in treating osteoarthritis. These therapies exhibit anti-inflammatory effects, improve function, and have the potential to regenerate cartilage. The study conducted on a rat model of chemically induced OA indicates that SVF treatment contributes to the histological maintenance of cartilage integrity and has an immunomodulatory action similar to ADSC (adipose-derived stem cell) treatment. Furthermore, it was observed that SVF treatment reduces the plasma levels of the inflammatory cytokine IL-1β [20]. Histological chondroprotective effects were also observed in surgically induced OA rat models treated with MFAT, explained by the downregulation of cartilage degradation enzymes (MMPs) and inflammatory factors (IL-6) [29]. In the mice model of chemically induced osteoarthritis, SVF treatment has a comparable anti-inflammatory effect to hADSC treatment. However, it has a less regenerative impact [19]. In the surgically induced mouse model of osteoarthritis, SVF treatment reduced cartilage-degenerative enzymes and inflammatory cytokines and increased macrophage polarization toward the M2 phenotype, thereby attenuating the progression of osteoarthritis [21]. The rabbit OA model studies observed the potential of SVF treatment to mitigate cartilage degeneration in affected joints by regulating chondrocyte viability, promoting a more favorable balance between anabolic and catabolic factors and activating M2 macrophages [27,28]. These studies also found MFAT treatment to be effective in promoting an anti-inflammatory environment. MFAT showed greater expression of CD-163 marker level, prolonged local biodistribution at the synovial membrane, and a structure that allows long-term survival of cells [26,27]. The studies reviewed only considered stromal vascular fraction treatment in the sheep osteoarthritis (OA) model. The proposed mechanisms of SVF to block OA progression were through colony-forming unit fibroblasts and stromal cell-associated characteristics. SVF showed little regenerative effect compared to ADSCs, which exhibited better cartilage matrix production [23]. SVF treatment showed superior effectiveness to ADSC treatment in counteracting an inflammatory microenvironment [25]. Levels of pro-inflammatory cytokines in synovial fluid exhibited decreased values in the SVF-treated group, with significantly lower levels of TNF-α and IL-6 compared to ADSC-treatment groups [22]. 

Several limitations need to be acknowledged in this review. Firstly, the observed heterogeneity in data reporting across the included studies poses a challenge to synthesizing findings. The variation in methodologies, outcome measures, and animal models used in these studies may impact the generalizability of the results. Encouraging a standardized approach in research studies can significantly reduce the heterogeneity in data reporting. Establishing consensus on key parameters, such as OA induction, injection procedures, assessment time points, and outcome measures, can enable more meaningful comparisons across studies. Additionally, the relatively limited number of studies available for review limits the ability to formulate definitive conclusions. This scarcity highlights the need for more comprehensive research in this field to establish robust patterns and trends. 

When choosing a method for inducing osteoarthritis in animal models, the advantages and disadvantages of each approach concerning the research objectives and translational relevance to human clinical outcomes should be carefully considered. By understanding the strengths and limitations of different in vivo models and induction methods, researchers can optimize the design and interpretation of their studies. Incorporating a variety of outcome evaluation methods ensures a comprehensive analysis of the effects of potential therapeutic interventions, ultimately advancing our understanding of osteoarthritis and enhancing the robustness of research findings.

## 5. Conclusions

This review provides a practical synthesis of the current state of research, highlighting areas where further investigation and standardization could advance our understanding of adipose tissue-derived injectable therapies in the context of osteoarthritis. Evaluating the possible outcomes of the minimally manipulated treatments synthesized in this review is necessary since they can serve as the basis for more advanced and effective therapies for the treatment of osteoarthritis.

## Figures and Tables

**Table 1:** Animal OA models and treatment characteristics.

Author, Year	Animal Model	Method of OA Induction	Groups and Types of Treatment	Therapy Administration Timing and Frequency after OA Induction	Period of Analysis
Muñoz-Criado et al., 2017 [19]	mouse	intra-articular injection of collagenase II with CaCl_2_	(1) control group (2) SVF-treated group (3) hADSC-treated group (4) PRP-treated group	once, at 5 days	4 weeks
Kamada et al., 2021 [21]	mouse	medial meniscotibial ligament resection	(1) control group (2) SVF-treated group	once, immediately	4 and 8 weeks
Ohashi et al., 2021 [29]	rat	anterior cruciate ligament, medial meniscus and medial collateral ligaments transection	(1) control group (2) MFAT-treated group	weekly, for six weeks	1 and 5 weeks
Yang et al., 2022 [20]	rat	intra-articular injection of sodium iodoacetate	(1) control group (2) SVF-treated group (3) ADSC-treated group	once, at 2 weeks	7 and 14 days
Kuroda et al., 2019 [28]	rabbit	anterior cruciate ligament transection	(1) control group (2) SVF-treated group	once, at 8 weeks	8 and 12 weeks
Desando et al., 2019 [27]	rabbit	anterior cruciate ligament transection	(1) MFAT-treated group (2) SVF-treated group (3) ADSC-treated group	once, at 8 weeks	1 and 4 weeks
Filardo et al., 2021 [26]	rabbit	anterior cruciate ligament transection	(1) control group (2) MFAT-treated group (3) SVF-treated group (4) ADSC-treated group	once, at 8 weeks	2 and 4 months
Lv et al., 2018 [23]	sheep	anterior cruciate ligament transection and medial meniscectomy	(1) control group (2) HA-treated group (3) low-dose ADSC (1 × 10^7^ ADSCs) + HA-treated group (4) high-dose ADSC (5 × 10^7^ ADSCs) + HA-treated group (5) SVF-treated group	twice, at 12 and 15 weeks	3 months
Veronesi et al., 2021 [22]	sheep	lateral meniscectomy	(1) control group (2) SVF-treated group (3) autologous 2.5 × 10^6^ ADSC-treated group (4) allogenic 2.5 × 10^6^ AESC-treated group	once, at 6 weeks	3 months
Veronesi et al., 2022 [25]	sheep	lateral meniscectomy	(1) control group (2) SVF-treated group (3) autologous 2.5 × 10^6^ ADSC-treated group (4) allogenic 2.5 × 10^6^ AESC-treated group	once, at 6 weeks	3 and 6 months
Berni et al., 2023 [24]	sheep	lateral meniscectomy	(1) control group (2) SVF-treated group (3) autologous 2.5 × 10^6^ ADSC-treated group (4) allogenic 2.5 × 10^6^ AEC-treated group	once, at 6 weeks	3 and 6 months

Abbreviations alphabetically ordered: ADSC: adipose-derived stem cell; hADSC: human adipose-derived stem cell; AEC: culture-expanded amniotic endothelial cell; AESC: culture-expanded amniotic epithelial stem cell; HA: hyaluronic acid; MFAT: mechanical micro-fragmented adipose tissue; SVF: stromal vascular fraction.

**Table 2:** Summary of relevant data extracted from the included studies evaluating minimally manipulated adipose tissue-derived injectable therapies.

Author, Year	Macroscopic Evaluation	Histologic Evaluation	Immunohistochemical Evaluation	Articular Biochemical Evaluation	Main Findings
Muñoz-Criado et al., 2017 [19]	NE	OARSI score	SOX-9	NE	chemically induced OA in the mice led to aggressive joint degeneration; significant increase in SOX-9 expression within the developing cartilage in the hADSC-treated group; endogenous induction of cartilage repair through hADSC injection rather than assuming cell replacement
Kamada et al., 2021 [21]	NE	OARSI score	collagen type II, MMP-13, ADAMTS-5, IL-6, IL-1β	NE	lower histological OARSI in the treated group compared to the control group; increased number of type II collagen-positive cells, decreased number of chondrocytes expressing MMP-13, ADAMTS-5, IL-6, and IL-1 in the treated group compared to the control group
Ohashi et al., 2021 [29]	descriptive evaluation	modified Mankin, histologic score	NE	NE	significantly lower modified Mankin histologic score in the treated group compared to the control group at 2- and 6-weeks evaluation
Yang et al., 2022 [20]	descriptive evaluation	observational	collagen type II, collagen type I	NE	both SVF and ADSC therapies exhibited, upon histological evaluation, a discernible thick layer of cartilage with a regular surface; both therapeutic interventions significantly reduced plasma IL-1β levels on days 7 and 14 after treatment compared to the control group
Kuroda et al., 2019 [28]	OARSI score	OARSI score	collagen type II, MMP-13, SOX-9	NE	macroscopically and histologically significantly lower OARSI scores and less cartilage damage in the treated group than the control group; immunohistochemically, the SVF group showed a significantly lower proportion of MMP-13-positive cells and a significantly higher percentage of type II collagen-positive areas than the control group; the proportion of SOX-9-positive cells was significantly higher in the SVF group than in the control group
Desando et al., 2019 [27]	NE	Laverty score, modified Pauli’s score	NE	NE	on day 7, SVF exhibited a significantly lower histologic scores compared to MFAT- and ASC-treated groups; on day 30, MFAT showed a significantly higher histologic score than SVF and ASCs; both SVF and MFAT groups displayed time-dependent effects on cartilage
Filardo et al., 2021 [26]	Hanashi score	Laverty score	collagen type II, MMP-1, TNF-α	NE	MFAT showed the best results both in terms of qualitative and semi-quantitative evaluations of articular cartilage, with a more uniform staining, a smoother surface and a significantly better Laverty score
Lv et al., 2018 [23]	ICRS score	cartilage thickness	collagen type X	IL-1β, IL-6	high-dose ADSC combined with HA significantly suppressed the expression of IL-1β and IL-6 in synovial fluid; there were no statistically significant differences observed in the treatment combinations of low-dose ADSC with HA and SVF with HA
Veronesi et al., 2021 [22]	gross articular damage score	NE	NE	IL-1β, CTX2, TNF-α, IL-6, PGE2	levels of pro-inflammatory cytokines (CTX2, PGE2, IL-1β) in synovial fluid, as assessed by ELISA, exhibited decreased values in the SVF-treated group; significantly lower levels of TNF-α and IL-6 were observed in both SVF- and AESC-treatment groups compared to the control and ADSC groups
Veronesi et al., 2022 [25]	gross articular damage score	OARSI score	collagen type II, collagen type I, MMP-13, IL-1β	IL-1β, CTX2, TNF-α, IL-6, PGE2	SVF treatment showed superior effectiveness compared to AESC treatment, which, in turn, exhibited superiority over ADSC treatment; SVF outperformed AESC concerning macroscopic scores at three months, Krenn scores at six months, histologic OARSI scores at three and six months; at both the 3-week and 6-week evaluation stages, the percentage of cells expressing COL1A1 and MMP-13 was significantly lower, while those expressing COL2A1 were significantly higher in the SVF-treatment group compared to AESC, ADSC, and control
Berni et al., 2023 [24]	gross articular damage score	cartilage thickness fibrillation index	collagen type II	IL-1β, CTX2, TNF-α, IL-6, PGE2	concerning articular cartilage viscoelastic parameters, notably the elastic modulus and cartilage relaxation time, SVF exhibited pronounced effectiveness at 3- and 6-months evaluation; comparative analysis with the other treatments revealed SVF’s superior success in attenuating the deterioration of articular cartilage viscoelastic parameters

Abbreviations alphabetically ordered: ADAMTS-5: disintegrin and metalloproteinase with thrombospondin motifs 5; COL1A1: Collagen Type I Alpha 1 Chain gene; COL2A1: Collagen Type II Alpha 1 Chain gene; CTX2: cross-linked C telopeptide of type II collagen; ICRS: International Cartilage Research Society; IL-1β: interleukin-1 beta; IL-6: interleukin-6; MMP-1: matrix metalloproteinase-1; MMP-13: matrix metalloproteinase-13; NE: not evaluated; OARSI: Osteoarthritis Research Society International’s cartilage histopathology assessment system; PGE2: prostaglandin E2; SOX-9: SRY-box transcription factor 9; TNF-α: tumor necrosis factor alpha.

## Data Availability

Not applicable.
